# Emergence and Global Spread of a Dengue Serotype 3, Subtype III Virus

**DOI:** 10.3201/eid0907.030038

**Published:** 2003-07

**Authors:** William B. Messer, Duane J. Gubler, Eva Harris, Kamalanayani Sivananthan, Aravinda M. de Silva

**Affiliations:** * University of North Carolina, Chapel Hill, North Carolina, USA; † Centers for Disease Control and Prevention, Fort Collins, Colorado, USA; ‡ University of California, Berkeley, California, USA; § Medical Research Institute, Colombo, Sri Lanka.

**Keywords:** Dengue, dengue hemorrhagic fever, dengue serotype 3, flavivirus, molecular epidemiology, virus Phylogeny, emerging virus, Sri Lanka, East Africa, Latin America, research

## Abstract

Over the past two decades, dengue virus serotype 3 (DENV-3) has caused unexpected epidemics of dengue hemorrhagic fever (DHF) in Sri Lanka, East Africa, and Latin America. We used a phylogenetic approach to evaluate the roles of virus evolution and transport in the emergence of these outbreaks. Isolates from these geographically distant epidemics are closely related and belong to DENV-3, subtype III, which originated in the Indian subcontinent. The emergence of DHF in Sri Lanka in 1989 correlated with the appearance there of a new DENV-3, subtype III variant. This variant likely spread from the Indian subcontinent into Africa in the 1980s and from Africa into Latin America in the mid-1990s. DENV-3, subtype III isolates from mild and severe disease outbreaks formed genetically distinct groups, which suggests a role for viral genetics in DHF.

Arthropod-borne viruses are responsible for the emergence of unexpected diseases in humans, as illustrated by the identification of West Nile virus encephalitis in the American hemisphere in 1999 (*1*). The emergence of a new disease is often attributable to the transport of a pathogen (as in the case of West Nile virus) or changes in the evolution or ecology of a native pathogen that hitherto caused mild or no disease in humans (*2*,*3*). We studied unexpected outbreaks of dengue hemorrhagic fever (DHF) in Sri Lanka, East Africa, and Latin America caused by dengue serotype 3 (DENV-3) virus.

Most persons infected with dengue viruses are asymptomatic or develop dengue fever (DF). DHF and dengue shock syndrome (DSS), which can be fatal, develop in a minority of infected persons. The pathogenesis of DHF is poorly understood, although factors such as age and previous exposure to dengue infections increase the risk for severe disease (*4*). Epidemiologic studies point to particular DENV strains being more virulent than others (*5*–*8*). For example, the dengue genotypes endemic to Central and South America have caused mild disease, while the Asian genotypes introduced to the region have led to DHF epidemics (*9*–*16*). Similarly, outbreaks of DHF in some Pacific islands have been traced to the introduction of Southeast Asian dengue strains (*17*). DENV-2 subtypes associated with mild and severe disease epidemics have distinct mutations in the E gene and 5′ and 3′ untranslated segments of the viral genome, although whether these mutations directly contribute to pathogenesis is unproven (*18*).

The distribution of DHF and DSS in Asia has been particularly puzzling. Before 1989, DHF was common in Southeast Asia but rare in the Indian subcontinent despite the circulation of all four serotypes in both regions. After 1989, this pattern of disease changed and regular epidemics of DHF were reported from several countries in the Indian subcontinent (*19*). Sri Lanka, in particular, experienced a dramatic and persistent increase in DHF cases (*20*). Epidemiologic studies of dengue in Sri Lanka have demonstrated that the intensity of virus transmission, as well as the relative abundance of each serotype, remained constant before and after the emergence of DHF (*21*). Thus, DHF did not emerge in Sri Lanka because of an overall increase in virus transmission or shift in serotype.

Although all four serotypes of dengue circulate in Sri Lanka, persons who have the severe form of the disease are most frequently infected with DENV-3 (*20*,*22*). Lanciotti et al. characterized the genetic relatedness of DENV-3 isolates from regions throughout the tropics and subtropics and identified four geographically distinct subtypes (*23*). All Sri Lankan isolates were classified as subtype III, which also includes isolates from East Africa and India, as well as recent isolates from Latin America. Because DENV-3 isolates from Sri Lanka isolated before and after 1989 (when DHF emerged) formed separate groups within subtype III, Lanciotti and colleagues postulated that a genetic shift in DENV-3 may have been responsible for the emergence of DHF (*23*).

In the current study, using phylogenetic methods, we analyzed DENV-3 viruses isolated from Sri Lanka for up to 10 years after the emergence of DHF to confirm the establishment of a new genotype and evaluate the roles of virus evolution and transport in establishing a new genotype. DENV-3, subtype III was introduced into Latin America in 1994 (*11*), and the virus has subsequently been isolated from DF and DHF outbreaks throughout Central and South America (*12*–*16*). We also examined the genetic relationships between DENV-3, subtype III isolates from Latin America, East Africa, and the Indian subcontinent. On the basis of our results, we describe the most likely scenario of events that led to the emergence of DENV-3–associated DHF in the Indian subcontinent and the Americas.

## Materials and Methods

### Virus Strains

The dengue virus strains sequenced for this study as well as sequences obtained from GenBank for this study are listed in Table 1. The virus isolates were obtained from the Centers for Disease Control and Prevention, Dengue Branch, Puerto Rico, and Division of Vector-Borne Infectious Diseases, Ft. Collins, Colorado; Medical Research Institute, Colombo, Sri Lanka; School of Public Health, Berkeley, California; Walter Reed Army Institute for Research, Washington, D.C.; and University of Massachusetts Medical Center, Worcester, Massachusetts.

**Table 1:** Dengue virus type 3 sequences used^a^

Strain	Y	Location	Name	Subtype	Sequence source	GenBank accession no.
D1266	1983	Sri Lanka	83SriLan1	III	This study	AF547225
D1306	1983	Sri Lanka	83SriLan2	III	This study	AF547226
D1307	1983	Sri Lanka	83SriLan3	III	This study	AF547227
D1336	1983	Sri Lanka	83SriLan4	III	This study	AF547228
D1440	1984	Sri Lanka	84SriLan1	III	This study	AF547229
073	1985	Sri Lanka	85SriLan	III	This study	AF547241
D2783	1989	Sri Lanka	89SriLan1	III	This study	AF547230
D2863	1989	Sri Lanka	89SriLan2	III	This study	AF547231
D2803	1989	Sri Lanka	89SriLan3	III	This study	AF547232
D3197	1990	Sri Lanka	90SriLan1	III	This study	AF547233
D5231	1993	Sri Lanka	93SriLan1	III	This study	AF547234
D9397	1994	Sri Lanka	94SriLan1	III	This study	AF547235
L57	1997	Sri Lanka	97SriLan1	III	This study	AF547242
K1	1998	Sri Lanka	98SriLan	III	This study	AF547243
1557	1985	Mozambique	85Mozamb1	III	This study	AF547236
1558	1985	Mozambique	85Mozamb2	III	This study	AF547237
1559	1985	Mozambique	85Mozamb3	III	This study	AF547238
251991	1991	Kenya	91Kenya	III	This study	AF547239
SOM079	1993	Somalia	93Somalia	III	This study	AF547240
32267	1994	Nicaragua	94Nicara1	III	This study	AF547244
6845	1998	Nicaragua	98Nicara1	III	This study	AF547245
7431	1998	Nicaragua	98Nicara2	III	This study	AF547246
7071	1998	Nicaragua	98Nicara3	III	This study	AF547262
BC 96/94	1994	Panama	94Panama1	III	This study	AF547247
032231	1994	Panama	94Panama2	III	This study	AF547248
BC 13/96	1994	Panama	94Panama3	III	This study	AF547249
BC 20/97	1996	Mexico	96Mexico1	III	This study	AF547250
BC 172/97	1996	Mexico	96Mexico2	III	This study	AF547251
BC 184/97	1996	Mexico	96Mexico3	III	This study	AF547252
BC173/97	1996	Mexico	96Mexico4	III	This study	AF547253
17605	1995	Costa Rica	95CostaR1	III	This study	AF547254
17608	1995	Costa Rica	95CostaR2	III	This study	AF547255
322473	1995	Costa Rica	95CostaR3	III	This study	AF547256
322488	1995	Costa Rica	95CostaR4	III	This study	AF547257
20/8	1997	Guatemala	97Guatem1	III	This study	AF547263
366-781	1998	Puerto Rico	98PuertoR1	III	This study	AF547258
400-996	2000	Puerto Rico	00PuertoR1	III	This study	AF547264
MK	1998	El Salvador	98ElSalv1	III	This study	AF547259
612210	2001	Venezuela	01Venezue1	III	This study	AF547260
VEN03	2001	Venezuela	01VEN03	III	This study	AF547261
Ref. 18	1981–91	Sri Lanka	81,85,89,91 SriLanA	III	GenBank	L11431,L11436–L11438
Ref. 18	1984	India	84IndiaA	III	GenBank	L11424
Ref. 18	1986	Samoa	86Samoa	III	GenBank	L11435
Ref. 18	1962–86	Thailand	62,73,86,86 Thailand	II	GenBank	L11440–L11442,L11620
Ref. 18	1983	Philippines	83Philipp	I	GenBank	L11432
Ref. 18	1989	Tahiti	89Tahiti	I	GenBank	L111619
Ref. 18	1992	Fiji	92Fiji	I	GenBank	L11422
Ref. 18	1973–85	Indonesia	73,78,85 Indones	I	GenBank	L11425,L11426,L11428
Ref. 18	1974–81	Malaysia	74,81 Malaysi	I	GenBank	L11429,L11427
Ref. 18	1956	Philippines	D3H-87	I	GenBank	L11423
Ref. 18	1963–77	Puerto Rico	63,77 PuertoR	IV	GenBank	L11433,L11434
Ref. 18	1965	Tahiti	65 Tahiti	IV	GenBank	L11439

^a^Includes original identifier for strain, year of isolation, taxa name used in this paper, dengue virus 3 subtype, source of viral sequence, and GenBank accession numbers.

### RNA Extraction

QiaAmp Viral RNA Mini Kit (QIAGEN, Valencia, CA) was used to extract viral RNA from both the mosquito grind supernatants and infected tissue culture media following the manufacturer’s protocol. Extracted RNA was stored at –70°C or immediately subjected to reverse transcription–polymerase chain reaction (RT-PCR).

### RT-PCR

DENV-3 RT-PCR was carried out as described by Lanciotti (23). Primers were designed to amplify and sequence a 966-bp fragment from positions 179–1,144, encompassing part of Capsid, all of PreM, and part of the E gene sequences. The reverse primer (DEN3/735) hybridized to positions 1,189–1,171 (5′-ctcctcaggcaaaaccgct-3′) and the forward primer (D1 consensus) hybridized to positions 132–159 (5′-tcaatatgctgaaacgcgcgagaaaccg-3′). The reverse primer DEN3/735 was added to extracted RNA, incubated at 85°C for 90 s, and allowed to cool to room temperature. RT was carried out for 45–60 min in 20 μL of reaction mix containing 25 U avian myeloblastosis virus reverse transcriptase (Roche, Nutley, NJ), deoxynucleoside tripophosphate, MgCl_2_, and RT buffer. PCR was performed by adding a 30-μL cocktail containing D1 consensus primer, PCR buffer, and EXPAND polymerase (Roche) to the 20-μL RT reaction. PCR conditions were 4 min at 94°C, 30–35 cycles of 94°C for 30 s, 54°C for 30 s, and 72°C for 90 s with 5 s/cycle added to elongation step after the first 10 cycles. We separated 5 μL of the reaction products on 2% agarose gels and visualized it by ethidium bromide staining. When necessary, target bands were excised and purified by using the Qiagen QIAquick Gel Extraction kit (QIAGEN) following manufacturer’s instructions. All remaining PCR reaction products were purified by using the Qiagen PCR Purification kit following the manufacturer’s protocol.

### DNA Sequencing

Purified PCR products were sent to the automated DNA sequencing facility at the University of North Carolina, Chapel Hill, NC. The DENV-3 sequences used in this manuscript included 40 newly determined sequences, which have been submitted to GenBank (accession nos. AF547225–AF547264).

### Viral Sequence Analysis

**Figure 1:**
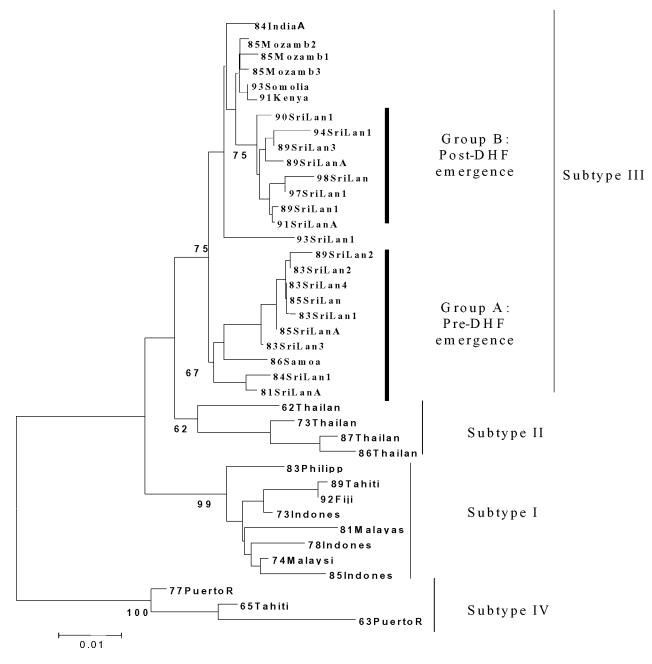
Phylogenetic tree of established dengue virus 3 subtypes (*23*) and the relationship of Sri Lanka pre– and post–dengue hemorrhagic fever dengue virus 3 (DENV-3) isolates to the established subtypes. This tree is based on a 708-base segment, positions 437 to 1145, spanning pre-M/M and a portion of the E gene. Scale bar shows number of substitutions per bases weighted by Tamura-Nei algorithm. Horizontal distances are equivalent to the distances between isolates. Numbers at nodes indicate bootstrap support values for the branch of the tree inferred at that node. The origin of the viruses and sequences used are listed in Table 1. The amino acid substitutions conserved within each DENV-3 subtype are listed in Table 2. DHF, dengue hemorrhagic fever.

**Figure 2:**
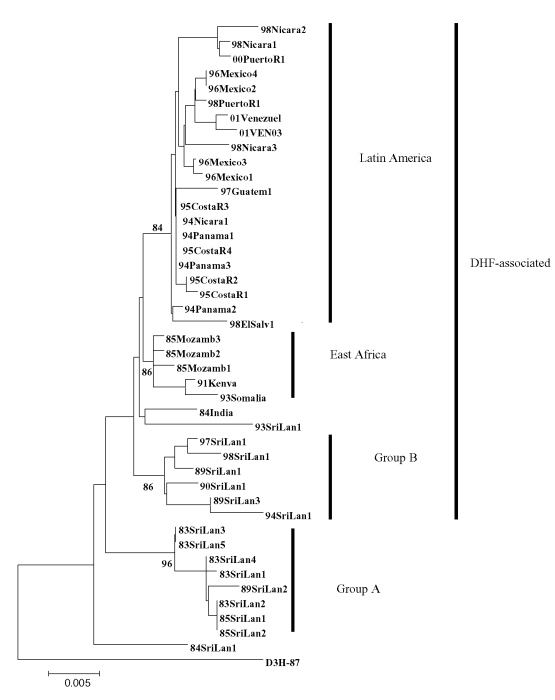
Phylogenetic tree of dengue virus 3, subtype III group A, group B, East Africa, and Latin America. Tree is based on 966-base region spanning positions 179–1,145 on the viral genome, capturing a portion of the C gene, all of pre-M/M gene and a portion of the E gene. Nucleotide substitutions conserved within each dengue virus 3, subtype III group (group A, B, East Africa, and Latin America) are listed in Table 3. DHF, dengue hemorrhagic fever.

Overlapping individual nucleic acid sequences were assembled with the aid of VECTOR NTI ContigExpress (InforMax, Inc., Bethesda, MD). Sequences were aligned and analyzed by using the following software: Clustal X (available from: URL: http://inn-prot.weizmann.ac.il/software/ClustalX.html), PAUP* (available from: URL: http://www.sinauer.com), PHYLIP (available from: URL: http://evolution.genetics.washington.edu/phylip.html), and MEGA II (available from: URL: http://www.megasoftware.net). Genetic distances were calculated by using Tamura-Nei distance algorithm with 1,000 bootstrap replicates; the trees were generated by using the Minimum Evolution method. The phylogenetic tree in Figure 1 is based on a 708-base segment, positions 437–1,145, spanning pre-M/M and a portion of the E gene. The phylogenetic tree presented in Figure 2 is based on 966-base region spanning positions 179–1,145 on the viral genome, capturing a portion of the C gene, all of pre-M/M gene, and a portion of the E gene.

**Table 2:** Amino acid substitutions conserved within each dengue virus 3 subtype for the isolates used to create the phylogenetic tree in Figure 1^a^

Subtype	Name	Position							
		31	55	57	128	135	148	188	234
Outgroup	56Philipp	I	H	T	L	I	L	D	I
I	73Indones	–	L	–	F	–	–	–	V
I	74Malaysi	–	L	–	F	–	–	–	V
I	78Indones	–	L	–	F	–	–	–	V
I	81Malaysi	–	L	–	F	–	–	–	V
I	83Philipp	–	L	–	F	–	–	–	V
I	85Indones	–	L	–	F	–	–	–	V
I	89Tahiti	–	L	–	F	–	–	–	V
I	92Fiji	–	L	–	F	–	–	–	V
II	62Thailan	–	–	A	–	–	W	–	–
II	73Thailan	–	–	A	–	–	–	–	–
II	86Thailan	–	L	A	–	–	–	–	–
II	87Thailan	–	L	A	–	–	–	–	–
III	85Mozamb1	–	–	–	–	–	–	–	–
III	85Mozamb2	–	–	–	–	–	–	–	–
III	85Mozamb3	–	–	–	–	–	–	–	–
III	84IndiaA	–	–	–	–	–	–	–	–
III	91Kenya	–	–	–	–	–	–	–	–
III	93Somolio	–	–	–	–	–	–	–	–
III	81SriLanA	–	–	–	–	–	–	–	–
III	83SriLan1	–	–	–	–	–	–	–	–
III	83SriLan2	–	–	–	–	–	–	–	–
III	83SriLan3	–	–	–	–	–	–	–	–
III	83SriLan4	–	–	–	–	–	–	–	–
III	84SriLan1	–	–	–	–	–	–	–	–
III	85SriLanA	–	–	–	–	–	–	–	–
III	85SriLan	–	–	–	–	–	–	–	–
III	89SriLan2	–	–	–	–	–	–	–	–
III	89SriLanA	–	–	–	–	–	–	–	–
III	89SriLan1	–	–	–	–	–	–	–	–
III	89SriLan3	–	–	–	–	–	–	–	–
III	90SriLan1	–	–	–	–	–	–	–	–
III	91SriLanA	–	–	–	–	–	–	–	–
III	93SriLan1	–	–	–	–	–	–	–	–
III	94SriLan1	–	–	–	–	–	–	–	–
III	97SriLan1	–	–	–	–	–	–	–	–
III	98SriLan1	–	–	–	–	–	–	–	–
III	86Samoa	–	–	–	–	–	–	–	–
IV	63PuertoR	T	–	–	F	L	M	E	–
IV	65Tahiti	T	–	–	F	L	M	E	–
IV	77PuertoR	T	–	–	F	L	M	E	–

^a^Reference strain 56Philipp is the highly passaged laboratory strain H87. Positions are numbered sequentially from the first position in the pre-M/M protein.

**Table 3:** Nucleotide substitutions conserved within dengue virus 3, subtype III groups^a^




Group	Strain	Position


338	429	503	566	653	686	695	707	728	734	749	791	821	866	896	902	978	1010	1019	1056

Reference	H87	A	A	C	G	G	T	T	G	C	C	C	A	T	C	T	T	C	C	T	C
Group A	83SriLan1	-	G	T	A	-	-	C	-	-	T	T	-	-	T	C	C	T	T	-	-
Group A	83SriLan2	-	G	T	A	-	-	C	-	-	T	T	-	-	T	C	C	T	T	-	-
Group A	83SriLan3	-	G	T	A	-	-	C	-	-	-	T	-	-	T	-	C	-	T	-	-
Group A	83SriLan4	-	G	T	A	-	-	C	-	-	T	T	-	-	T	C	C	T	T	-	-
Group A	84SriLan1	-	G	-	-	-	-	-	-	-	-	T	-	-	-	C	-	-	-	-	-
Group A	85SriLan1	-	G	T	A	-	-	C	-	-	T	T	-	-	T	C	C	T	T	-	-
Group A	89SriLan2	-	G	T	A	-	-	C	-	-	T	T	-	-	T	C	C	T	T	-	-
unclassified	84India	G	G	-	-	A	-	-	-	-	-	T	-	-	-	-	-	-	-	G	-
unclassified	93SriLan1	G	G	-	-	A	-	-	-	-	-	-	-	-	-	-	-	-	-	G	-
East Africa	85Mozamb1	G	-	-	-	A	-	-	-	-	-	-	-	-	-	-	-	-	-	G	-
East Africa	85Mozamb2	G	-	-	-	A	-	-	-	-	-	-	-	-	-	-	-	-	-	G	-
East Africa	85Mozamb3	G	-	-	-	A	-	-	-	-	-	-	-	-	-	-	-	-	-	G	-
East Africa	91Kenya	G	-	-	-	A	-	-	-	-	-	-	-	-	-	-	-	-	-	G	-
East Africa	93Somalia	G	-	-	-	A	-	-	-	-	-	-	-	-	-	-	-	-	-	G	
Group B	89SriLan1	G	G	-	-	A	-	-	A	T	-	-	-	C	-	-	-	-	-	G	-
Group B	89SriLan3	-	G	-	-	A	-	-	A	T	-	-	-	C	-	-	-	-	-	G	-
Group B	90SriLan1	G	G	-	-	A	-	-	A	T	-	-	-	C	-	-	C	-	-	G	-
Group B	94SriLan1	-	G	-	-	A	-	-	A	T	-	-	-	C	-	-	-	-	-	G	-
Group B	97SriLan1	G	G	-	-	A	-	-	A	T	T	-	-	C	-	C	-	-	-	G	-
Group B	98SriLan1	G	G	-	-	A	-	-	A	T	-	-	-	C	-	C	-	-	-	G	-
L. America	94Nicara1	G	G	-	-	A	C	-	-	-	-	-	G	C	-	-	-	-	-	G	T
L. America	94Panama1	G	G	-	-	A	C	-	-	-	-	-	G	C	-	-	-	-	-	G	T
L. America	94Panama2	G	G	-	-	A	C	-	-	-	-	-	G	C	-	-	-	-	-	G	T
L. America	94Panama3	G	G	-	-	A	C	-	-	-	-	-	G	C	-	-	-	-	-	G	T
L. America	CostaRica1	G	G	-	-	A	C	-	-	-	-	-	G	C	-	-	-	-	-	G	T
L. America	CostaRica2	G	G	-	-	A	C	-	-	-	-	-	G	C	-	-	-	-	-	G	T
L. America	CostaRica3	G	G	-	-	A	C	-	-	-	-	-	G	C	-	-	-	-	-	G	T
L. America	CostaRica4	G	G	-	-	A	C	-	-	-	-	-	G	C	-	-	-	-	-	G	T
L. America	96Mexico1	G	G	-	-	A	C	C	-	-	-	-	G	C	-	-	-	-	-	G	T
L. America	96Mexico2	G	G	-	-	A	C	-	-	-	-	-	G	C	-	-	-	-	-	G	T
L. America	96Mexico3	G	G	-	-	A	C	-	-	-	-	-	G	C	-	-	-	-	-	G	T
L. America	96Mexico4	G	G	-	-	A	C	-	-	-	-	-	G	C	-	-	-	-	-	G	T
L. America	97Guatem1	G	G	-	-	A	C	-	-	-	-	-	G	C	-	-	-	-	-	G	T
L. America	98Nicara1	G	G	-	-	A	C	-	-	-	-	-	G	C	-	-	-	-	-	G	T
L. America	98Nicara2	G	G	-	-	A	C	-	-	-	-	-	G	C	-	-	-	-	-	G	T
L. America	98Nicara3	G	G	-	-	A	C	-	-	-	-	-	G	C	-	-	-	-	-	G	T
L. America	98ElSalv1	G	G	-	-	A	C	-	-	-	-	-	G	C	-	-	-	-	-	G	T
L. America	01Venezue1	G	G	-	-	A	C	-	-	-	-	-	G	C	-	-	-	-	-	G	T
L. America	01VEN03	G	G	-	-	A	C	-	-	-	-	-	G	C	-	-	-	-	-	G	T
L. America	98PuertoR1	G	G	-	-	A	C	-	-	-	-	-	G	C	-	-	-	-	-	G	T
L. America	00PuertoR1	G	G	-	-	A	C	-	-	-	-	-	G	C	-	-	-	-	-	G	T


^a^Reference strain is the highly passaged laboratory strain H87. Positions are numbered sequentially from the first nucleotide position at the 5¢ end of the genome.

## Results and Discussion

Many investigators have used viral nucleotide sequence data and phylogenetic methods to understand genetic relationships between viruses, as well as the epidemiology of viral disease. Phylogenetic studies have shown that dengue viruses can move long distances between continents (*24*) as well as short distances between neighboring countries (*25*). Our goal was to use a phylogenetic approach to understand recent DHF outbreaks caused by DENV-3 infections in the Indian subcontinent and Latin America.

Previous phylogenetic analysis of DENV-3 has principally relied on complete or partial sequences of the pre-M/M and E genes (*13*,*16*,*17*,*23*). Our analysis used a 708-base segment, positions 437–1,145, spanning pre-M/M and a portion of the E gene, coding for 236 amino acids. This region was selected because it both conserved the original phylogenetic relationship identified by Lanciotti et al. and, in preliminary analysis with previously established sequences, captured 44% of the variable sites within DENV-3, subtype III Sri Lankan sequences. No insertion/deletion mutations and no hypervariable regions were detected in this span.

A total of 40 DENV-3 sequences, including 21 sequences available from GenBank and 19 newly determined Indian subcontinent and African sequences (Table 1) were compared. Dates of isolation ranged from 1963 to 1998. With the exception of 63PuertoR, all sequences were from low-passage (<4) virus cultures. Several approaches to phylogenetic analysis, including maximum likelihood, parsimony, and distance methods, were compared. All approaches yielded identical or nearly identical topologies. Results presented here used the Tamura-Nei algorithm to calculate genetic distances and the minimum evolution method to create the trees (Figure 1). The tree identifies four distinct lineages that correspond to the region of isolation, reproducing the same evolutionary relationship first described by Lanciotti, et al. (*23*). Subtype I includes isolates from Southeast Asia and the South Pacific islands; subtype II consists of isolates from Thailand; subtype III is comprised of isolates from the Indian subcontinent, East Africa, and a single isolate from Samoa; and subtype IV includes Puerto Rico and Tahiti. Similarity within subtypes was high, with subtype III showing the greatest mean similarity (98.4%), followed by subtypes I, II, and IV (Table 4).

**Table 4:** Summary of within- and between-subtype nucleotide mean similarity for dengue virus 3 isolates shown in Figure 1^a^

Subtype	Within subtype similarity *(*%)	Between subtype similarity (%)		
		I	II	III
I	98.1			
II	97.7	94.9		
III	98.4	95.6	96.3	
IV	97.6	92.3	92.5	92.7

^a^Mean similarities were calculated with the Tamura-Nei distance algorithm.

All 24 Sri Lankan, Indian, and East African strains fell into subtype III (Figure 1). The circulating virus genotypes within this region have remained closely related over the relatively long period of 18 years (1981–1998), indicating that countries bordering the western Indian Ocean form a geographically distinct region with regard to DENV-3 viruses. DENV-2 viruses in the regions also form a subtype with a similar geographic distribution (*26*,*27*). Frequent trade between East Africa, Western Indian Ocean islands, and the Indian subcontinent may have been responsible for the movement of dengue viruses throughout the region (*26**,**28*). Rico-Hesse, for example, demonstrated the introduction of DENV-2 to Africa from islands in the Indian Ocean (26). The earliest subtype III virus on record is an isolate from India in 1966; this virus occupies a node that is ancestral to all the subsequent Asian and African isolates (R.S. Lanciotti, pers. comm.), suggesting that the DENV-3, subtype III viruses have their origin in the Indian subcontinent and have subsequently spread out of the region.

In Sri Lanka, regular epidemics of DHF have been observed only since 1988. DENV-3 is responsible for many of the infections that progress to DHF (*20*,*22*). DENV-3 isolates obtained before and after the emergence of DHF are very closely related and belong to subtype III, indicating that the emergence of DHF on the island is not due to the introduction of a new subtype from outside the region. However, within subtype III, most Sri Lankan isolates (except for 93SriLan1) from before and after the emergence of DHF segregated into two distinct clades, designated groups A and B (Figure 1). Group A, with nine isolates from 1981 to 1989, consists of viruses collected up to the year epidemic DHF emerged in Sri Lanka but contains no isolates from later than 1989. Group B includes eight isolates from 1989 to 1998 but none from before 1989. Temporally, the two groups are continuous, by virtue of sharing isolates in 1989. Group A includes isolate 89SriLan2, while group B contains 89SriLan1, 89SriLan3, and 89SriLanA. However, the groups do not form a continuous lineage; they share a common ancestor only at the node for subtype III (Figure 1). Group B shares ancestral nodes with isolates from India and East Africa. Because the Indian and East African isolates overlap temporally with group A (all isolates are from the 1980s), group A and group B lineages likely diverged sometime before 1981 and followed distinct evolutionary pathways.

We propose two likely scenarios that led to the emergence of group B viruses in Sri Lanka. One possibility is that the group B viruses were introduced from India or East Africa into Sri Lanka because Indian and East African isolates from the mid-1980s are closely related to Sri Lankan group B viruses (Figure 1). Of the two regions, India is the more likely source because of geographic proximity to Sri Lanka, although the East African viruses could be the direct ancestors of the group B viruses. Another possibility is that both groups co-circulated in Sri Lanka in the early 1980s, with group B being a minor population. Some selective force operating in the late 1980s may have shifted the balance in favor of group B viruses. In either case, group B viruses emerged in Sri Lanka because a subtype III variant already established in the greater region became more common in Sri Lanka and not because a novel virus evolved and emerged de novo on the island.

**Figure 3:**
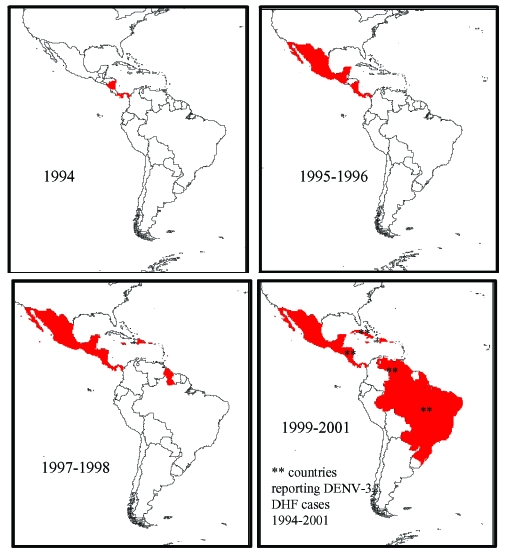
Map of the spread of dengue virus 3 (DENV-3), subtype III through Latin America and the Caribbean. The introduction of DENV-3, subtype III was first reported in November 1994 in Nicaragua and Panama. This virus strain has been isolated, identified, and reported in at least 16 other countries in the region. *Represents countries with dengue hemorrhagic fever (DHF) caused by DENV-3. These countries are Nicaragua in 1994 and 1998, Brazil and Venezuela in 2001 (Pan American Health Organization, unpub. data).

DENV-3, subtype III was detected in the Americas during DF and DHF outbreaks in Nicaragua and Panama in 1994 (*11*). Subsequently, the virus has spread to many countries in Latin America, and DENV-3–associated DHF was confirmed in several countries (*13*,*14*,*16*,*29*–*31*) (Figure 3). To establish the relationship of recent Latin American DENV-3 isolates to each other and to the previously identified Indian subcontinent and East African subtype III isolates, we sequenced and analyzed a 966-base region spanning positions 179–1,145 on the viral genome, capturing a portion of the C gene, all of pre-M/M gene, and a portion of the E gene. This region adds 288 positions to the 5′ end of the sequences initially presented in this study.

Forty-three isolates were sequenced (21 from Mexico and Central and South America, 16 from Sri Lanka, 1 from India, and 5 from East Africa) (Table 1). The D3H-87 belonging to DENV-3, subtype 1 was used as an outgroup. Years of isolation ranged from 1983 to 2001, an 18-year span. Except for the D3H-87 outgroup, all other isolates were low-passage clinical isolates. Most of the nucleotide mutations were silent: only 12 amino acid positions showed any variability and only 2 positions showed variability in more than one isolate. Consequently, the evolutionary relationships observed in this analysis likely reflect the results of genetic drift and are unlikely to have been influenced by host-specific selection events on this portion of the genome (*26*).

All sequences included in this analysis fell within subtype III (data not shown). Several approaches to phylogenetic analysis were compared, and all approaches yielded identical or nearly identical topologies. We used the Tamura-Nei algorithm to calculate genetic distances and the minimum evolution method to create the trees (Figure 2). Bootstrap values are shown at critical nodes. Despite the high overall similarity of the isolates in this analysis, geographically and temporally distinct groups formed separate lineages. Generally, two separate lineages formed within subtype III. The first consists of group A viruses isolated from 1981 to 1989 in Sri Lanka. These viruses have been associated only with DF. The second is composed of Sri Lankan group B, Indian, East African, and all of the isolates from Mexico and Central and South America.

Within group A, members are closely related, with a nucleotide mean similarity of 99.4% (Table 5). Within the expanded group B and related viruses, three distinct clades exist: a group of closely related Sri Lankan isolates from 1989 to 1998, 5 East African isolates from 1985 to 1993, and 21 isolates from 1994 to 2001 from Latin America. Isolates 84India and 93SriLan1 are less closely related to the other geographically distinct isolates in the larger second lineage.

**Table 5:** Summary of within- and between-group nucleotide mean similarity for the dengue virus 3, subtype III virus isolates shown in Figure 2^a^

Subgroup	Within-group similarity	Between group similarity		
		Subgroup A	East Africa	Subgroup B
Subgroup A	99.4%			
East Africa	99.5%	98.2%		
Subgroup B	98.8%	97.9%	98.7%	
Latin America	99.5%	98.0%	99.0%	98.5%

^a^Mean similarities were calculated with the Tamura-Nei distance algorithm.

The isolates from Latin America all emerge from a common node on the tree, suggesting a single introduction of a virus and the subsequent diversification of the virus population from the founding strain. The DENV-3, subtype III isolates from Nicaragua, Panama, and Costa Rica are closest to the Latin American group’s originating node, with the more recent isolates found farther from that node, reflecting the viral population’s ongoing evolution after the point source introduction.

**Figure 4:**
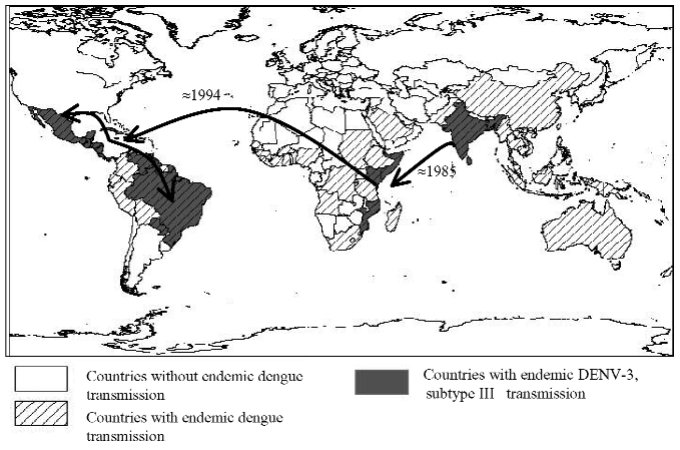
Global spread of dengue virus 3 (DENV-3), subtype III, which has been continuously circulating in the Indian subcontinent from the 1960s to the present. The virus was first isolated from East Africa in 1985 in Mozambique and subsequently from Kenya (1991) and Somalia (1993) (*32*,*33*). DENV-3 subtype III was first detected in the American continent in 1994 (Nicaragua and Panama) and the virus has subsequently spread through most of Latin America (*13*,*14*,*16*,*29*,*30*). The arrows depict the most likely directions of spread based on the phylogenetic relationships between the viruses (see text for details). The map also displays countries in which dengue is known to occur.

The internal branch from the Latin American group shares a common node with the isolates from East Africa. The common hypothetical ancestor for Latin America and East Africa then shares a common node with the Sri Lankan group B virus isolates. Both on the phylogenetic tree and in pair-wise comparisons (Table 5), the Latin American group was more closely related to the isolates from East Africa than to the group B Sri Lankan isolates. Furthermore, the East African isolates pre-date the earliest Latin American isolates by 9 years, while the less closely related Sri Lankan group B isolates are nearly contemporaneous with the Latin American isolates. Therefore, the point source DENV-3 introduction into Latin America is most likely to have its origins in East Africa and not the Indian subcontinent (Figure 4).

Little is known about dengue activity in Africa, particularly DENV-3 (3*2*). DENV-3 was first detected on the African continent in 1984 to 1985 during an outbreak in Mozambique (*32*). Later studies of U.S. troops in Africa and the Persian Gulf suggested that DENV-3 is endemic in those regions but largely undetected (*33*). Our results show that all East African DENV-3 isolates belong to subtype III. The fact that DENV-3 was only first isolated from East Africa in 1985, whereas the viruses were present in the Indian subcontinent at least as far back as 1966 (R.S. Lanciotti, pers. comm.), suggests that DENV-3, subtype III was introduced from the Indian subcontinent into East Africa in or before 1984 (Figure 4). This introduction led to the establishment of a stable East African group of DENV-3, subtype III because all the isolates from Mozambique, Kenya, and Somalia isolated from 1985 to 1993 form a distinct clade within subtype III (Figure 3).

The DENV-3, subtype III viruses introduced into Latin America are most closely related to subtype III viruses in East Africa (Figure 3). Although we can only speculate about the exact mode of transport of DENV-3 into Latin America, we propose that Panama, with its canal that attracts goods as well as civilians and military personnel from other parts of the world, may have been the point of introduction of subtype III into the Americas. Similarly, the introduction of DENV-2 in 1981 into Cuba may be attributable to Cuban military personnel traveling between Southeast Asia and Cuba (*24*,*34*).

Epidemiologic and clinical studies on dengue in Indonesia in the 1970s pointed to strain differences between DENV-3 viruses contributing to transmission and disease severity (*35*,*36*). Despite their overall similarity at the nucleotide level, the DENV-3, subtype III isolates examined in this study have been associated with severe or mild disease outbreaks (Figure 3). Sri Lankan group A viruses were isolated during a time of little to no DHF, while group B viruses were isolated after the emergence of DHF in Sri Lanka. The emergence of DHF in Sri Lanka was not accompanied by a change in dengue transmission or the abundance of any particular serotype (*21*). Implicating DENV-3 directly as the cause of DHF in Sri Lanka has been difficult because few virus isolates are available from DHF patients in Sri Lanka. However, during dengue surveillance studies in 1997, only DENV-3 was isolated from hospitalized dengue cases, whereas DENV-1, DENV-2, and DENV-3 were isolated from patients visiting outpatient clinics (*22*). These observations suggest that DENV-3 is responsible for severe dengue disease in Sri Lanka. Further studies are required to better establish the relative contribution of DENV-3 to severe disease in Sri Lanka.

The current studies support a viral genetic basis for severe and mild disease outbreaks. We found that the population of DENV-3 viruses associated with DHF in Sri Lanka did not appear to be direct descendants of the group A viruses that were circulating before DHF emerged in that country. The Sri Lankan 1989–1997 isolates are more closely related to the isolates from East Africa and the isolates from the Americas than they are to the isolates from 1981 to 1989 in Sri Lanka (Figure 3). All three groups of subtype III viruses (Sri Lankan group B, East African group, and Latin American group) associated with DHF are more closely related to each other than they are to the pre–DHF group A viruses from Sri Lanka (Figure 3). Thus, all the viruses within subtype III are closely related (mean 98.4% identity at the nucleotide level), yet they form distinct phylogenetic groups associated with mild or severe disease.

The Sri Lankan group B viruses may be associated with severe disease unlike group A viruses because the group B viruses are inherently more virulent. Alternatively, the ability of preexisting dengue antibody to neutralize group A viruses and enhance group B viruses may account for the observed associations with severe and mild disease. In a recent study, antibodies against American DENV-1 viruses neutralized the Native American DENV-2 genotype better than the Southeast Asian DENV-2 genotype that is currently circulating in the Americas and causing DHF (*37*). This study lends support to the idea that Asian DENV-2 may produce a more severe disease not because of inherent virulence properties but because persons with previous primary DENV-1 infections may enhance infection with this genotype and neutralize infections with the Native American DENV-2 genotype. Similarly, DENV-2 and -3 are the common serotypes in Sri Lanka, and persons with previous primary DENV-2 infections could neutralize the DENV-3 group A viruses better than the group B viruses. This difference may explain the unexpected emergence of DHF associated with group B. Further comparative studies with group A and B viruses are needed to understand their association with mild and severe disease, respectively.
